# Exploring the anti-gastric cancer mechanisms of Diosgenin through integrated network analysis, bioinformatics, single-cell sequencing, and cell experiments

**DOI:** 10.3389/fphar.2025.1600960

**Published:** 2025-05-23

**Authors:** Zhangjun Yun, Qianru Yang, Chengyuan Xue, Yang Shen, Liyuan Lv, Suicai Mi, Li Hou

**Affiliations:** ^1^ Department of Oncology and Hematology, Dongzhimen Hospital, Beijing University of Chinese Medicine, Beijing, China; ^2^ Graduate School of Beijing University of Chinese Medicine, Beijing, China; ^3^ Department of Oncology, Xiamen Hospital, Beijing University of Chinese Medicine, Xiamen, China

**Keywords:** gastric cancer, Diosgenin, network analysis, plk1, MDM2, p53, mechanism

## Abstract

**Background:**

To comprehensively investigate the mechanism of action of Diosgenin elements against gastric cancer (GC).

**Methods:**

Targets of Diosgenin were collected from six databases, and enrichment analysis was used to identify its associated diseases and biological pathways. GC-related genes were identified using weighted gene co-expression network analysis. A multi-approach strategy, including network analysis, bioinformatics, single-cell RNA sequencing, Mendelian randomization, and cell experiments, was used to explore the anti-GC mechanisms of Diosgenin.

**Results:**

In this study, 605 Diosgenin targets were identified, with key involvement in cell apoptosis, TNF signaling, and platinum resistance pathways, demonstrating significant enrichment in GC. Diosgenin may exert its anti-GC effects through 311 targets, involving regulation of the cell cycle, p53, and FoxO signaling pathway. Key effectors, including CDK1, CCNA2, TOP2A, CHEK1, and PLK1, were identified. Single-cell sequencing indicated that TOP2A, HSP90AA1, and HSP90AB1 might be crucial immune regulatory targets of Diosgenin. Diosgenin significantly inhibited GC cell proliferation, colony formation, migration, and invasion. Evidence from western blot analysis indicates that Diosgenin exerts anti-GC effects by suppressing the expression of PLK1 and MDM2 proteins while upregulating p53 protein levels.

**Conclusion:**

These findings highlight Diosgenin’s potential as a promising therapeutic agent for GC, offering a foundation for future research and clinical applications.

## 1 Introduction

Gastric cancer (GC) ranks as the fifth most common malignancy and the fourth leading cause of cancer-related death globally in 2020 (Bray et al., 2024). Due to the subtle and nonspecific nature of early symptoms, most GC patients are diagnosed at an advanced stage, resulting in poor prognosis. Although various treatment modalities, including surgery, radiotherapy, chemotherapy, immunotherapy, and targeted therapy, have improved the efficacy to some extent, the overall therapeutic outlook for GC patients remains bleak, particularly due to the issue of multi-drug resistance (MDR) in later stages (Wei et al., 2020). Moreover, chemotherapy induces numerous specific side effects in cancer patients, such as gastrointestinal reactions, nephrotoxicity, myelosuppression, and neurotoxicity, which significantly impair their quality of life (Oun et al., 2018). Thus, promising strategies for GC treatment should involve identifying novel drugs that are highly effective in inhibiting cancer cell growth while exhibiting minimal adverse effects.

Natural products are a valuable source of compounds with novel chemical structures that are both effective and less toxic (Sethi et al., 2018). Notably, one-third of all new drugs approved by the United States Food and Drug Administration (US FDA) are derived from natural products and their derivatives (Sethi et al., 2018). Particularly in the field of cancer, from the 1940s to the end of 2014, 49% of the 175 approved small molecule drugs were either natural products or directly derived from them (Newman and Cragg, 2016). Diosgenin, a bioactive metabolite derived from plants of the Dioscoreaceae family, such as D. nipponica and D. panthaica Prain et Burk, has been a key metabolite of traditional herbal medicine in China since the 1960s, particularly in the context of cancer treatment. Several studies have shown that Diosgenin exhibits diverse biological activities, including lowering lipid levels, reducing inflammation, inhibiting cell proliferation, lowering blood sugar, and acting as a potent antioxidant (Jesus et al., 2016). Additionally, Diosgenin has been found to inhibit cancer cell proliferation and induce apoptosis in various cancer cell lines, such as those of gastric (Gu et al., 2021), colorectal (Li et al., 2021), hepatocellular (Li et al., 2010), breast (Khanal et al., 2022), and osteosarcoma (Khanal et al., 2022). In particular, regarding GC, our team previously discovered that Diosgenin may exert anti-proliferative effects on GC cells by regulating the expression of Bcl-2, Akt proteins, and the genes caspase 3, E2F1, and E2F3 (Li et al., 2019). Additionally, other studies have claimed that Diosgenin can resist the proliferation and invasion of GC cells in a low-oxygen environment (Mao et al., 2012), and also inhibit the proliferation of GC cells by suppressing the expression of MESP1 (Gu et al., 2021) and regulating the expression of cell adhesion molecules (Mao et al., 2012) in GC cells. Research by Liu et al. revealed that Diosgenin induces significant G0/G1 cell cycle arrest and apoptosis in GC cells (Liu et al., 2020). Furthermore, Diosgenin, in combination with GSK126, can exert a stronger inhibitory effect on GC cell proliferation by downregulating epithelial-mesenchymal transition (EMT)-related molecules through the inhibition of the Rho/ROCK signaling pathway (Liu et al., 2020). Thus, it is reasonable to hypothesize that Diosgenin has significant potential in the prevention and treatment of GC by modulating multiple targets, pathways, and biological processes. Given the multifaceted biological alterations in cancer and the early-stage research on the specific targets and pathways of Diosgenin’s anti-GC effects, a systematic analysis of its pharmacological mechanisms is highly necessary.

Network pharmacology, a rapidly growing subfield of pharmacology, has become essential in modern drug development (Li et al., 2024). Moving beyond the traditional “one drug, one target, one disease” approach, network pharmacology emphasizes the modulation of multiple targets and pathways, providing a nuanced understanding of drug actions *in vivo* (Nogales et al., 2022). This approach allows for a comprehensive analysis of drug interactions within biological systems, enhancing predictions of therapeutic outcomes and potential side effects. Integrating network analysis with bioinformatics, single-cell sequencing, and molecular docking techniques is crucial in new drug development. Bioinformatics supplies the data and analytical tools needed to build and interpret complex biological networks, while single-cell sequencing can identify the expression status of drug targets in individual cells, aiding in a more detailed understanding of disease cellular heterogeneity and complex biological processes. Molecular docking aids in simulating drug-target protein interactions, allowing for the prediction of ligand-receptor affinity.

**FIGURE 1:**
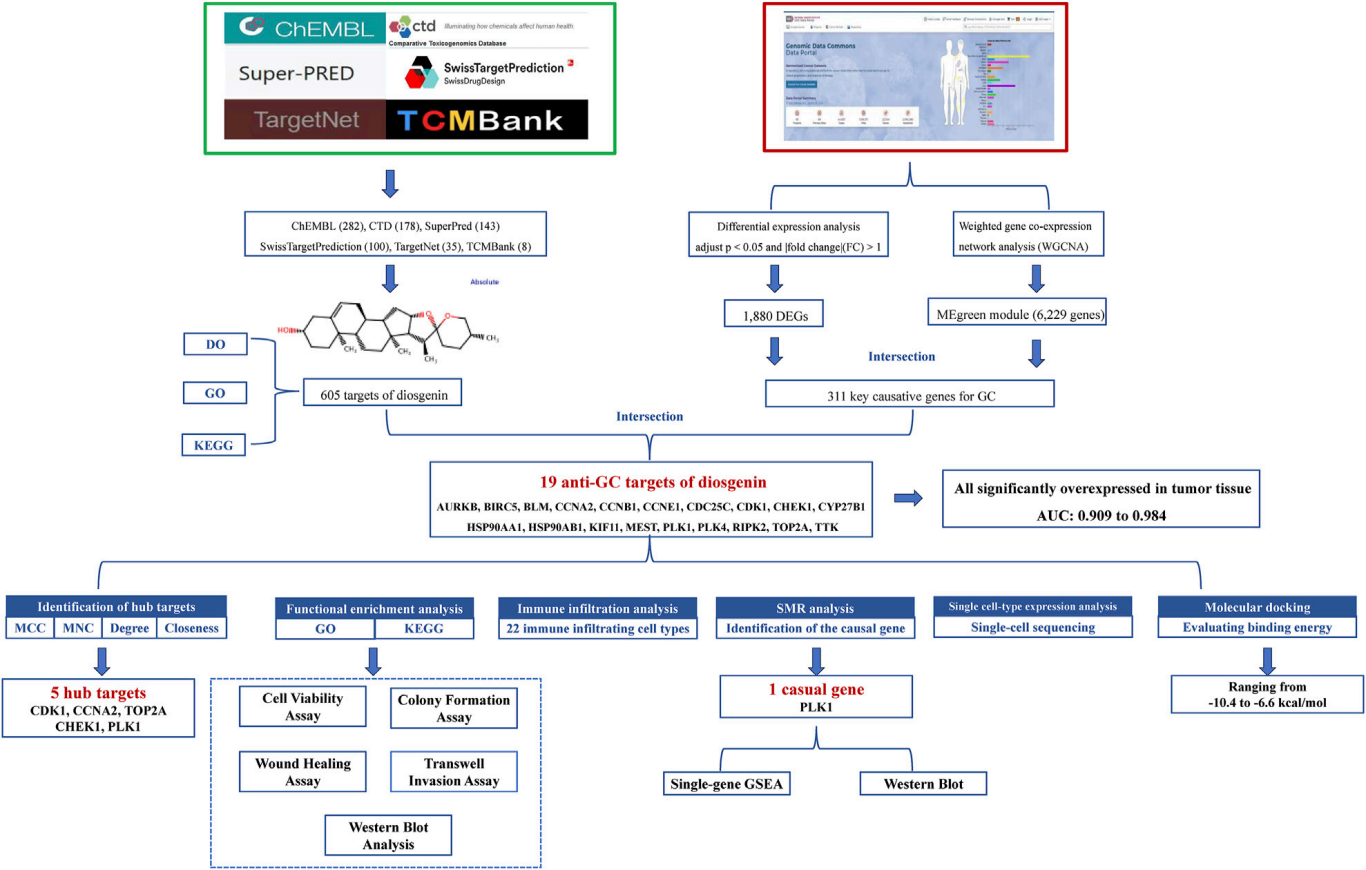
Elucidating the anti-GC mechanisms of diosgenin: a comprehensive flowchart approach.

Thus, this work comprehensively investigates the anti-GC mechanisms of Diosgenin by integrating network analysis, bioinformatics, single-cell sequencing, molecular docking and cell experiments. The goal of this research is to establish a solid theoretical foundation for the clinical application of Diosgenin. The detailed workflow of this study is shown in Figure 1.

## 2 Materials and methods

### 2.1 Identification of potential targets and biological functions of Diosgenin

Potential targets of Diosgenin were accessed from ChEMBL https://www.ebi.ac.uk/chembl/), CTD (http://ctdbase.org/), SuperPred (https://prediction.charite.de/), SwissTargetPrediction (http://swisstargetprediction.ch), TargetNet (http://targetnet.scbdd.com/home/index/), and TCMBank (https://www.tcmbank.cn/) databases. The functional enrichment analyses for potential targets of Diosgenin including Disease Ontology (DO), Gene Ontology (GO), and Kyoto Encyclopedia of Genes and Genomes (KEGG) were performed (Wu et al., 2021; Yu et al., 2015). The threshold for significant enrichment was set at q value <0.05.

### 2.2 WGCNA identified key causative genes for GC

The transcriptome profile analysis of GC was obtained from The Cancer Genome Atlas (TCGA) (https://portal.gdc.cancer.gov/), containing 412 GC tissue samples and 36 healthy tissue samples (TCGA-STAD). First, the raw data were normalized using the ‘Limma 3.58.1’ package of the R 4.3.3 software, and then the following criteria were adopted to identify the differentially expressed genes (DEGs) in GC: adjust p < 0.05 and |fold change|(FC) > 1. Next, we performed Weighted gene co-expression network analysis (WGCNA) (Langfelder and Horvath, 2008) on the gene expression data of GC to capture the gene modules strongly associated with the development of GC. Finally, we identified key modules by measuring the association of gene modules with normal and GC through gene significance (GS) values and module membership (MM) values. We intersected the key modular genes identified by WGCNA with differentially expressed genes taken to finally identify key causative genes for GC.

### 2.3 Functional enrichment analysis

The targets of Diosgenin and GC-causing genes were intersected to determine the target of anti-GC effect of Diosgenin. Similarly, we also performed GO and KEGG enrichment analyses on these targets to assess the biological characteristics of Diosgenin anti-GC. Subsequently, the STRING v12.0 database (https://string-db.org/) was applied to construct a protein-protein interaction (PPI) network (medium confidence = 0.4) to assess the interactions between the proteins encoded by the above-mentioned genes. Utilizing Cytoscape 3.10.1 software, we further optimized the PPI network and employed four widely used algorithms within the cytoHubba plugin—Matthews Correlation Coefficient (MCC), Maximum Neighborhood Component (MNC), Degree, and Closeness—to identify central nodes within the network.

### 2.4 Immune infiltration analysis

The CIBERSORT algorithm estimates the proportions of various immune cell types based on the expression levels of immune-related genes (Newman et al., 2015). Gene expression matrices for 22 infiltrating immune cell types were obtained from the CIBERSORTX database (https://cibersortx.stanford.edu/). Using the CIBERSORT algorithm, the correlations between the anti-GC targets of Diosgenin and the expression of these 22 immune infiltrating cell types were investigated using a non-parametric correlation method (Spearman).

### 2.5 Differential expression and diagnostic efficacy

For the anti-GC targets of Diosgenin, the differential expression of these targets in GC and normal tissues in the TCGA-STAD dataset was examined using the Wilcoxon rank-sum test. The ‘pROC 1.18.5’ package of R 4.3.3 was applied to plot the receiver operating characteristic (ROC) curve, and the area under curve (AUC) was calculated to evaluate the diagnostic efficacy of the targets (Grau et al., 2015).

### 2.6 SMR analysis and single-gene GSEA

Mendelian randomization (MR) studies utilize genetic variations as instrumental variables (IVs) to infer causality between exposure and outcome, offering insights into whether observed associations reflect causal relationships (Davies et al., 2018). Since genetic variations are randomly assigned at conception, MR analysis effectively reduces confounding bias. Moreover, MR mitigates the risk of reverse causation because genetic variations are established prior to the onset of disease. Thus, we obtained the cis expression quantitative trait loci (eQTLs) for the anti-GC targets of Diosgenin from the eQTLGen Consortium (31,684 blood samples) (Vosa et al., 2021) and the GTEx Consortium V8 (670 blood samples; https://www.gtexportal.org/home/) as IVs, and genome-wide association studies (GWAS) data of GC (1,423 cases and 314,193 controls) from the FinnGen consortium (Kurki et al., 2023) as outcome. Using Summary-data-based Mendelian randomization (SMR) analysis, we quantified the causal relationships between these targets and GC. Subsequently, tumor samples in the TCGA-STAD dataset were divided into high and low expression cohorts based on the median expression levels of the identified causal genes. Single-gene gene set enrichment analysis (single-gene GSEA) was performed, referencing the KEGG pathway database, to evaluate the regulatory pathways of the causal genes in GC with a Normalized Enrichment Score (NES)| > 1, adjusted p value <0.05.

### 2.7 Single cell-type expression analysis

The cell type-specific expression of intersecting targets was further evaluated by using single-cell RNA-seq data (GSE183904) from human GC tissues and adjacent normal tissues from the Gene Expression Omnibus (GEO) of Kumar et al. (Kumar et al., 2022). Kumar et al. performed quality control of single-cell data based on the following criteria: each sample was considered for genes/features shared by three or more cells, and cells showing 500 or more features and fewer than 6,000 features. Also, cells with mitochondrial RNA percentages of >20 were filtered out. Subsequently, we performed NormalizeData, ScaleData, RunPCA, and RunHarmony functions on the data by using the “Seurat 5.0.0”package (Butler et al., 2018) in the R language. Set afPC to 20, resolution to one and apply tsne for dimensionality reduction clustering. The FindAllMarkers function was used to identify markers in each cell type and the ‘SingleR 2.4.1’ package (Aran et al., 2019) was applied to annotate the cell types. Differential gene expression was compared on each cell type in GC and normal tissues under the guidelines of min.pct = 0.25 and logfc.threshold = 0.25.

### 2.8 Molecular docking

To further elucidate the binding affinity and interaction pattern between Diosgenin (ligand) and the targets (receptors), molecular docking simulations were performed in this study. Utilizing Autodock Vina 1.5.7 (Morris et al., 2008), a sophisticated computational tool for protein-ligand docking, we executed docking studies involving Diosgenin and proteins encoded by 13 selected genes. Drug structural data of Diosgenin (PubChem ID:99474) was retrieved from the PubChem Compound Database (https://pubchem.ncbi.nlm.nih.gov/), while protein structural information was obtained from the Protein Data Bank (PDB, https://www1.rcsb.org/). A grid box was centered to encapsulate each protein domain, allowing unhindered molecular flexibility during docking. With a spacing of 1.0 Å and dimensions set to 40 × 40 × 40 Å^3^ to define the docking pocket, ensuring comprehensive exploration of the binding site. The resultant docking poses were visually inspected and analyzed using PyMOL version 2.6.

### 2.9 Cell culture

The Human GC cell lines AGS was purchased from China EallBio Biomedical Technology Co., Ltd. (bio-72964, EallBio China, Beijing, China). This cell line, originally isolated in 1979 from the gastric tissue of a 54-year-old Caucasian female diagnosed with gastric adenocarcinoma, exhibits epithelial morphology. AGS cells were cultured in Ham’s F-12 medium supplemented with 10% fetal bovine serum and 1% penicillin/streptomycin. The cells were maintained in a humidified incubator at 37°C with 5% CO2.

### 2.10 Cell viability assay

Diosgenin (HY-N0177, MCE China, Shanghai, China) was dissolved in dimethyl sulfoxide (DMSO) and subsequently diluted in complete culture medium before being applied to treat GC cells. Cell viability was assessed using the Cell Counting Kit-8 (CCK-8, D5879, Beyotime China, Shanghai, China), which was purchased from China Beyotime Biomedical Technology Co., Ltd. AGS cells in the logarithmic growth phase were seeded into 96-well plates at a density of 5,000 cells/mL and incubated for 24 h to ensure proper cell attachment. Subsequently, the cells were treated with Diosgenin at concentrations of 0, 5, 10, 15, 20, 30, and 60 µM in complete culture medium, with each concentration tested in six replicate wells. After 24, 48, and 72 h of treatment, 20 µL of CCK-8 solution was added to each well, and the plates were incubated at 37°C for at least 30 min under light-protected conditions followed by thorough mixing. The absorbance (OD value) of each well was measured at a wavelength of 480 nm using a microplate reader. Based on the OD values, cell viability curves were plotted to assess the dose- and time-dependent effects of Diosgenin on AGS cells.

### 2.11 Colony formation assay

AGS cells in the logarithmic growth phase were seeded into six-well plates at a density of 7 × 10^2^ cells/mL. The control group was treated with complete culture medium, while the treatment group received Diosgenin. The experiment was terminated when colonies became visible to the naked eye. Colonies were fixed with 4% paraformaldehyde and stained with 0.1% crystal violet. Colony counts were performed using ImageJ software.

### 2.12 Wound healing assay

AGS cells in the logarithmic growth phase were seeded into six-well plates at a density of 5 × 10^5^ cells/well. After 24 h, a scratch was introduced, and the cells were incubated with low-serum medium for the control group or low-serum medium containing Diosgenin for the treatment group. Images were captured at 0 and 24 h, and wound closure was quantified using ImageJ software.

### 2.13 Transwell invasion assay

Transwell inserts were coated with 60 µL of diluted Matrigel and incubated at 37°C for 3 h to form a thin film. Excess liquid was removed, and 100 µL of serum-free medium was added to each insert for 30 min to hydrate the membrane. AGS cells were seeded into the upper chamber at a density of 4 × 10^5^ cells/mL in serum-free medium (control) or serum-free medium containing Diosgenin (treatment). Complete medium was added to the lower chamber. After 24 h of incubation, cells were fixed with 4% paraformaldehyde, stained with 0.1% crystal violet, and photographed. Invaded cells were counted using ImageJ software.

### 2.14 Western blot analysis

Total proteins were extracted using RIPA lysis buffer containing protease and phosphatase inhibitors. Protein concentration was measured using the BCA assay, followed by electrophoresis and transfer to a membrane. The membrane was blocked with a rapid blocking buffer for 30 min, incubated with primary antibodies at 4°C overnight, and then with secondary antibodies at room temperature for 90 min. Protein bands were visualized using ECL chemiluminescence. Antibodies targeting MDM2 (86934S, 1:1,000), p53 (2524T, 1:1,000), anti-mouse IgG (7074P2, 1:1000), and anti-rabbit IgG (7076P2, 1:1000) were purchased from Cell Signaling Technology (CST) in Massachusetts, United States. The PLK1 antibody (F0393) was obtained from Selleck Chemicals in Wuhan, China. The glyceraldehyde 3-phosphate dehydrogenase (GAPDH) antibody (60004-1-Ig, 1:50,000) was procured from Proteintech in Chicago, United States.

### 2.15 Statistical analysis

Experimental data were analyzed using GraphPad Prism 8 and SPSS 25.0 software. Data were expressed as mean ± standard deviation (SD). Comparisons among multiple groups were performed using one-way ANOVA, and comparisons between two groups were conducted using the t-test. A p value of <0.05 was considered statistically significant.

## 3 Results

### 3.1 Identification and evaluation of potential targets for Diosgenin

**FIGURE 2:**
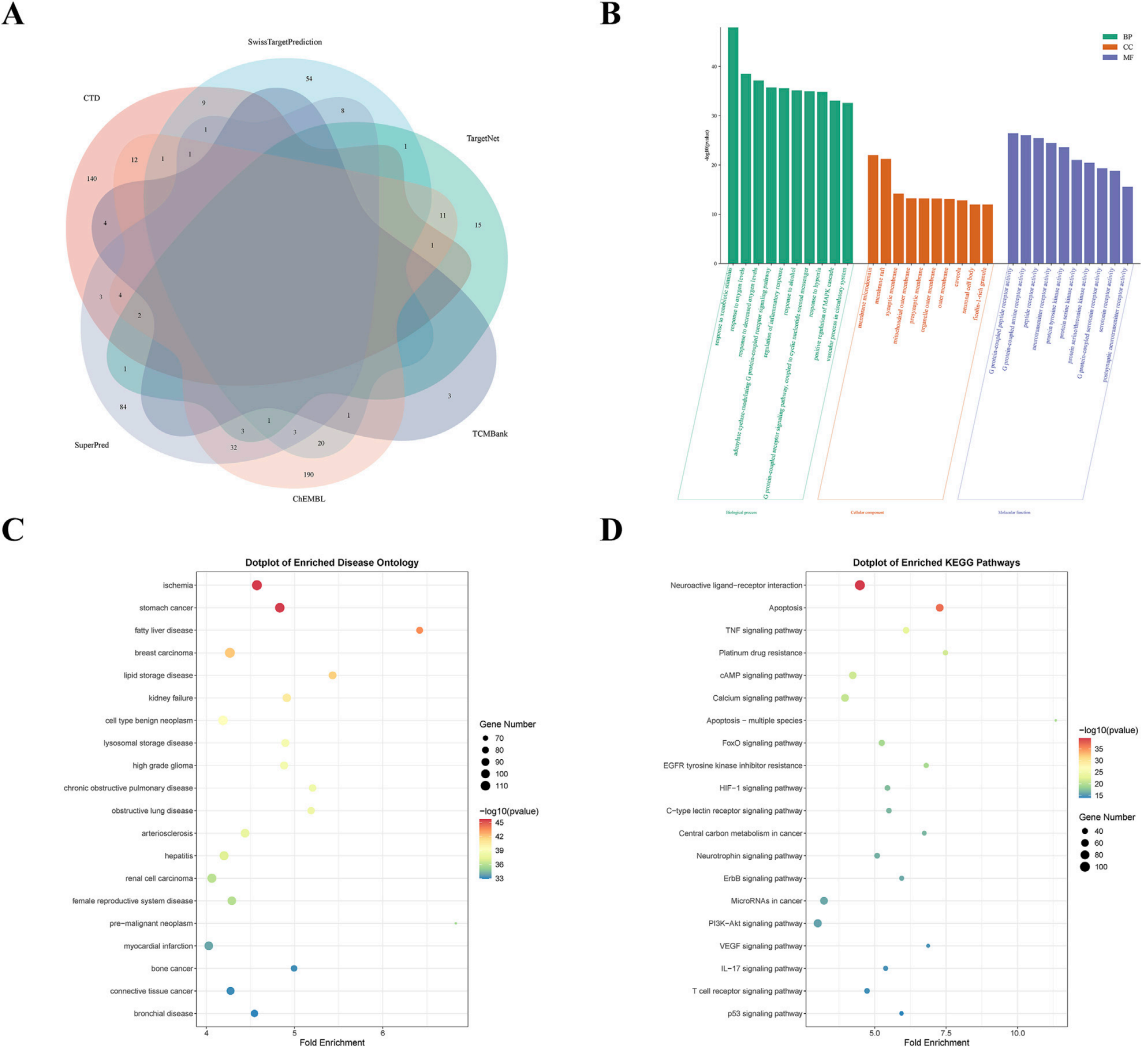
Screening analysis of Diosgenin targets. **(A)** Venn diagram of Diosgenin in the six databases. **(B)** Gene Ontology (GO) enrichment analysis (BP, biological process; CC, cellular component; MF, molecular function) of Diosgenin targets. **(C)** Disease Ontology (DO) enrichment analysis of Diosgenin targets. **(D)** Kyoto Encyclopedia of Genes and Genomes (KEGG) enrichment analysis of Diosgenin targets.

A total of 746 potential targets for Diosgenin were identified from the ChEMBL (282), CTD (178), SuperPred (143), SwissTargetPrediction (100), TargetNet (35), and TCMBank (8) databases. After removing duplicates, 605 unique targets were obtained (Figure 2A). GO enrichment analysis (Figure 2B) revealed that these targets were primarily involved in biological processes (BP) such as response to decreased oxygen levels, regulation of inflammatory response, and positive regulation of MAPK cascade. They were significantly enriched in cellular components (CC) including membrane microdomain, membrane raft, and the mitochondrial outer membrane, encompassing molecular functions (MF) like G protein-coupled peptide receptor activity, peptide receptor activity, and neurotransmitter receptor activity. Notably, DO enrichment analysis revealed that the targets of Diosgenin action were significantly enriched in GC (Figure 2C). KEGG enrichment analysis revealed that Diosgenin was mainly involved in neuroactive ligand-receptor interactions, apoptosis, TNF signaling pathway, platinum resistance, cAMP and p53 signaling pathways (Figure 2D).

### 3.2 Critical causative genes for GC identified by WGCNA

**FIGURE 3:**
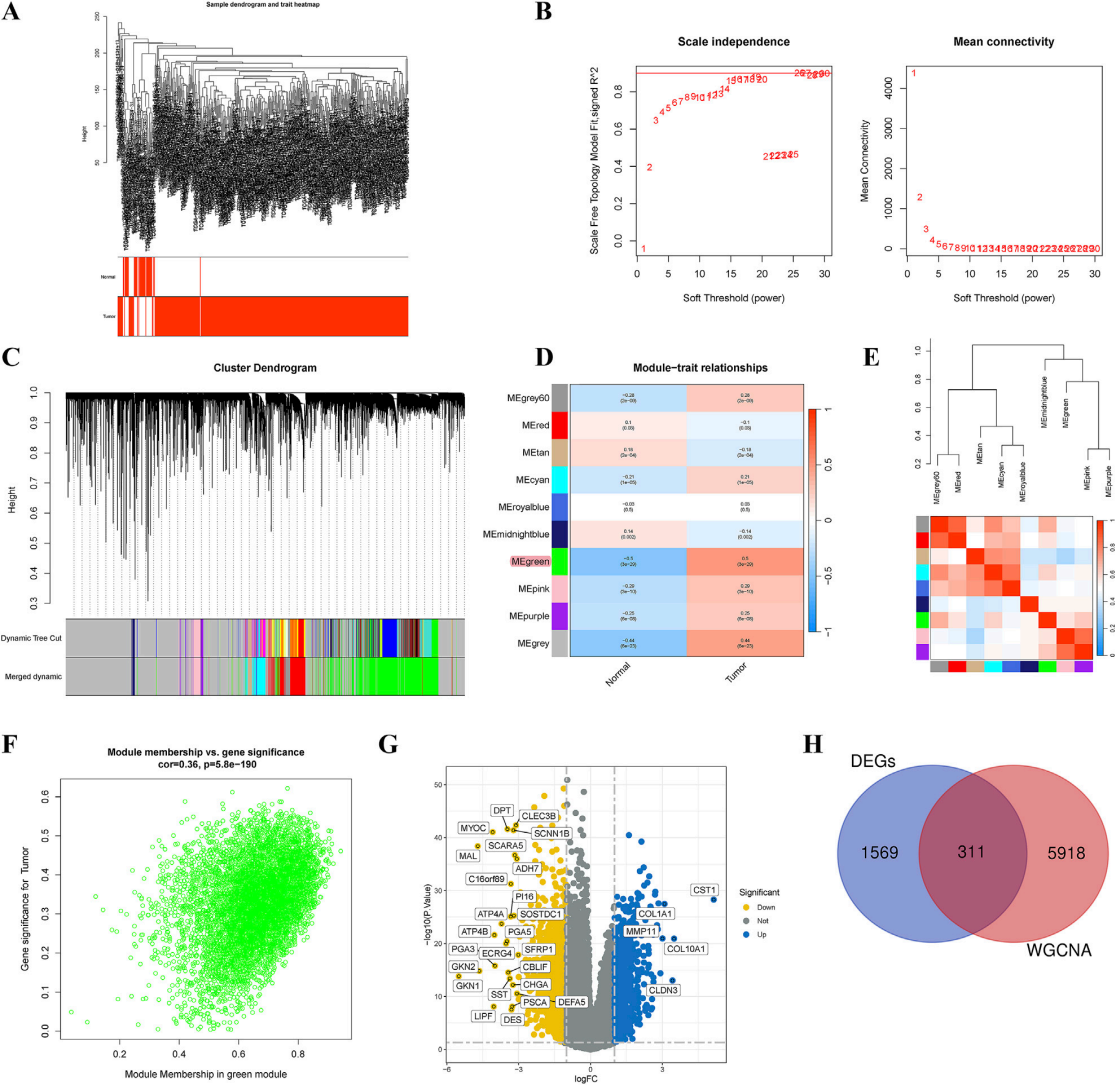
Identification of critical causative genes in gastric cancer through gene co‐expression network analysis (WGCNA). **(A)** Sample dendrogram and trait heatmap. **(B)** Selection of soft thresholds. **(C)** Cluster dendrogram of WGCNA. **(D)** Correlations between gene modules and melanoma status. **(E)** Correlation between modules. **(F)** Correlation between brown module memberships and gene significance. **(G)** A volcano plot presented the differentially expressed genes (DEGs) in gastric cancer. **(H)** Venn diagram showed the intersection genes of genes identified by WGCNA and DEGs.

We utilized microarray data from TCGA-STAD to perform WGCNA. Outlier detection indicated no significant outliers in the data (Figure 3A). The soft-thresholding power was assessed at 20, achieving a scale-free index of 0.9, suggesting reasonable network connectivity (Figure 3B). Subsequently, the TOM and adjacency matrix among genes were constructed. A co-expression network was then established, and a cluster dendrogram was generated using dynamic tree cutting and merging dynamic modules (Figure 3C). Ultimately, the clustering results partitioned the data into 11 modules (Figure 3D). We calculated the correlation coefficients between each module and GC-related phenotypes. The results indicated that the MEgreen module exhibited the strongest correlation with GC (COR = 0.50, p = 3E-29). Figure 3E presented the heatmap of module-trait relationships. The scatter plot of MM and GS within the MEgreen module showed a high correlation (COR = 0.36, p = 5.8E-190) (Figure 3F). Thus, the MEgreen module (6,229 genes) could be the optimal module for elucidating GC phenotypes. Additionally, differential expression analysis identified 1,880 DEGs (Figure 3G). By intersecting the genes from the MEgreen module with these DEGs, we identified the 311 key causative genes for GC (Figure 3H).

### 3.3 Functional enrichment and immune infiltration analysis

**FIGURE 4:**
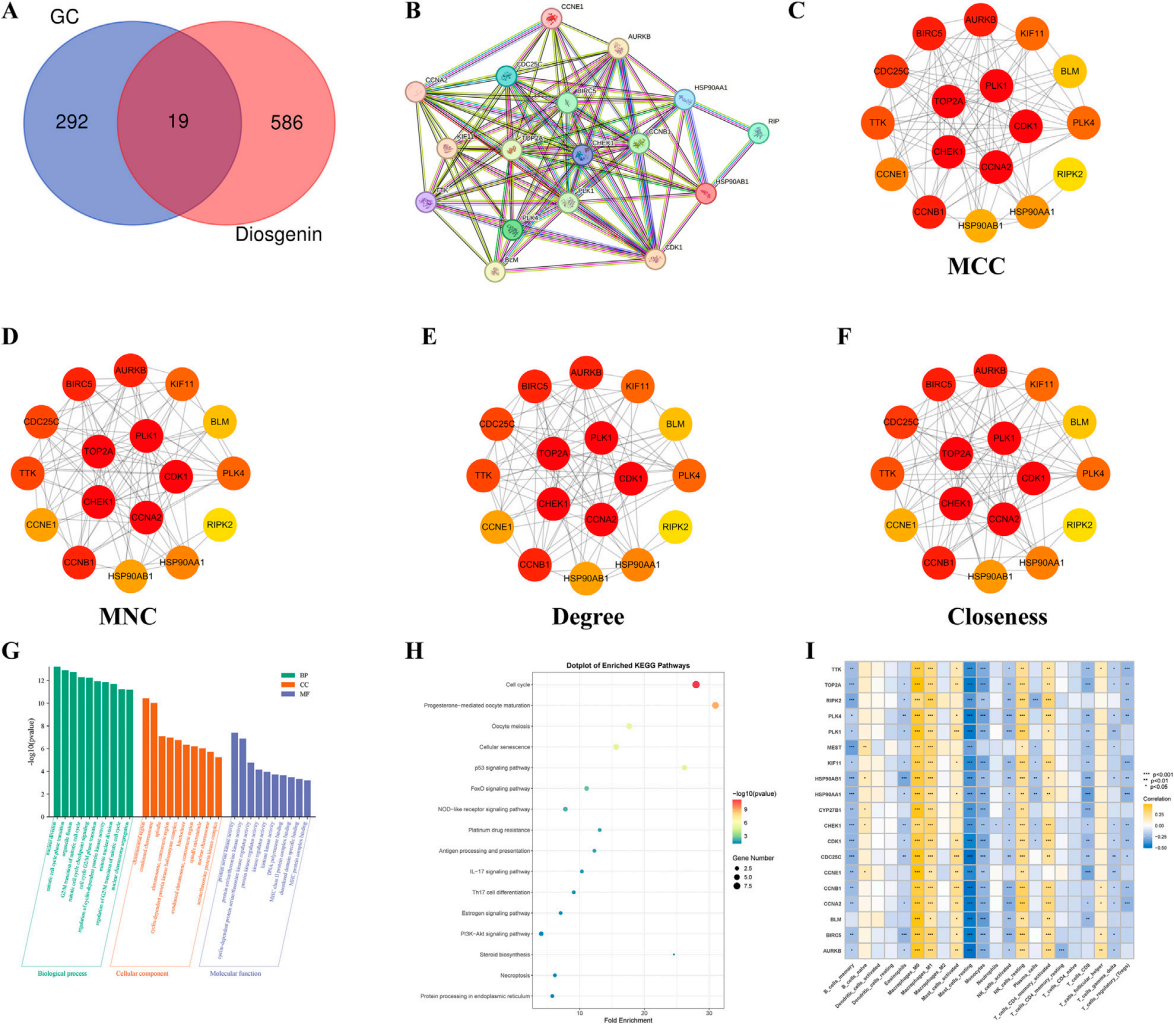
Functional enrichment and immune infiltration analysis for anti-gastric cancer targets. **(A)** Venn diagram illustrated the intersection genes of Critical causative genes in gastric cancer and targets of Diosgenin. GC: gastric cancer. **(B)** Construct a protein-protein interaction network of Diosgenin in the treatment of gastric cancer using the STRING database.** (C–F) **The network plot displayed the importance of each gene evaluated using four algorithms (Matthews Correlation Coefficient (MCC), Maximum Neighborhood Component (MNC), Degree, and Closeness) in Cytoscape 3.10.1. Darker colors of nodes indicate greater importance of the gene. **(G)** Gene Ontology (GO) enrichment analysis (BP, biological process; CC, cellular component; MF, molecular function) of anti-gastric cancer targets. **(H)** Kyoto Encyclopedia of Genes and Genomes (KEGG) enrichment analysis of anti-gastric cancer targets. **(I)** Heatmap illustrated the correlation between anti-gastric cancer targets and 22 immune cells.

The intersection of 605 Diosgenin targets with 311 key pathogenic genes for GC yielded 19 potential anti-GC targets of Diosgenin (Figure 4A). These intersecting targets were imported into the STRING database to construct a PPI network (19 nodes, 104 edges, average node degree of 10.9, p value <1.0E-16). The results revealed strong interactions among the proteins encoded by 17 genes (Figure 4B). Notably, consensus across four algorithms consistently identified CDK1, CCNA2, TOP2A, CHEK1, and PLK1 as significant nodes in the PPI network (Figures 4C–4F). GO enrichment analysis demonstrated that Diosgenin exerts its anti-GC effects primarily through regulating the cell cycle, acting on chromosomal region, and modulating protein serine/threonine kinase activity (Figure 4G). KEGG enrichment analysis (Figure 4H) showed that Diosgenin anti-GC targets were significantly enriched in pathways related to cell cycle, cellular senescence, p53, FoxO, NOD-like receptor, and platinum drug resistance. Immune infiltration analysis (Figure 4I) revealed that the expression of Diosgenin anti-GC targets is significantly positively correlated with macrophage M0 and M1, resting NK cells, and activated memory CD4^+^ T cells, while being significantly negatively correlated with memory B cells, resting mast cells, monocytes, and activated NK cells.

### 3.4 Differential expression and diagnostic efficacy

**FIGURE 5:**
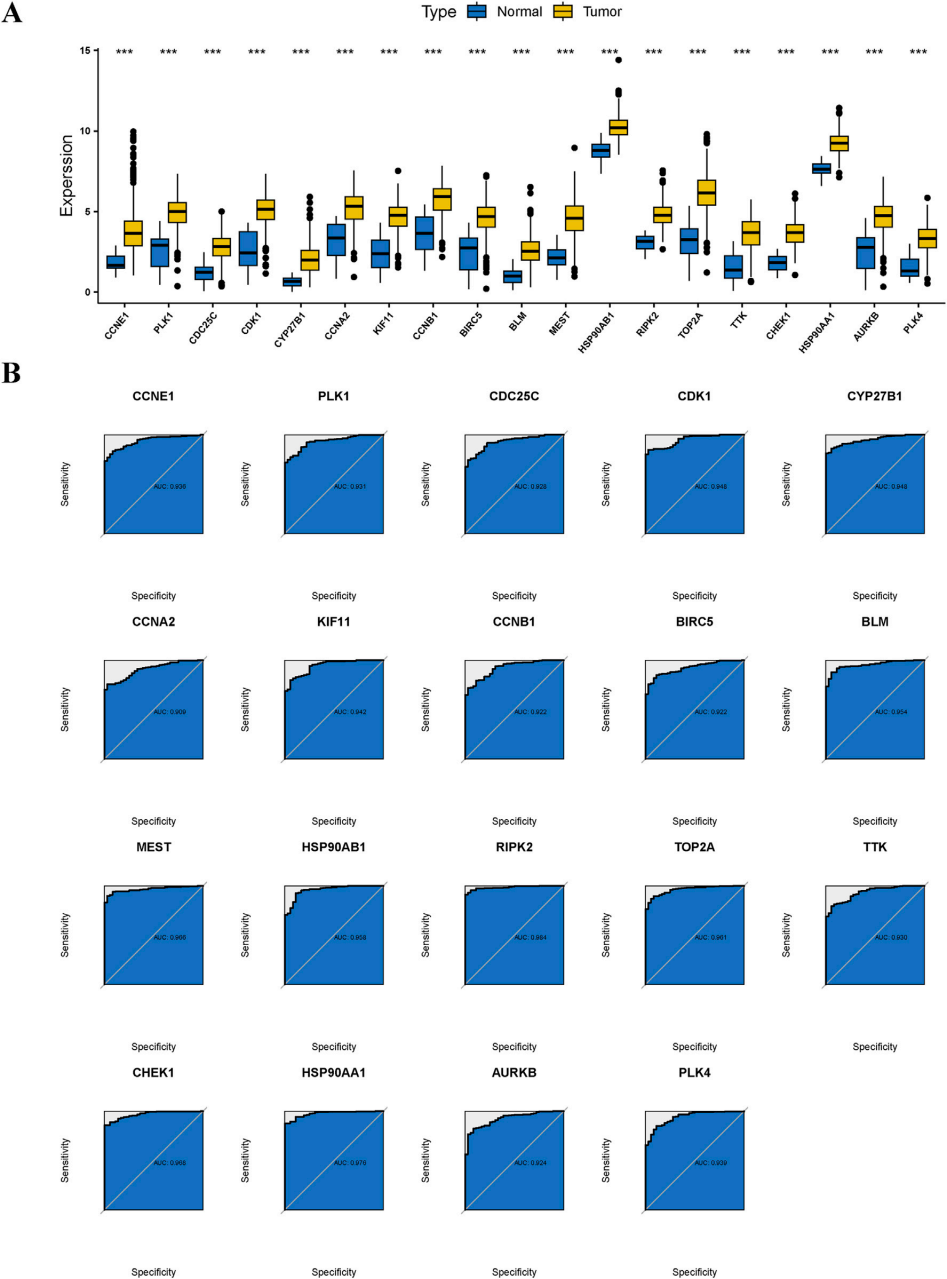
Differential expression and diagnostic efficacy for anti-gastric cancer targets. **(A)** Box plots showed the differential expression of anti-gastric cancer targets in tumor tissues and normal tissues. P value < 0.001***. **(B)** Receiver operating characteristic illustrated the diagnostic efficacy of anti-gastric cancer targets.

Evidence from the Wilcoxon rank-sum test indicated that the 19 anti-GC targets of Diosgenin were significantly overexpressed in GC tissues (Figure 5A). ROC curves showed excellent clinical discrimination of these targets (Figure 5B).

### 3.5 SMR analysis and single-gene GSEA

**FIGURE 6:**
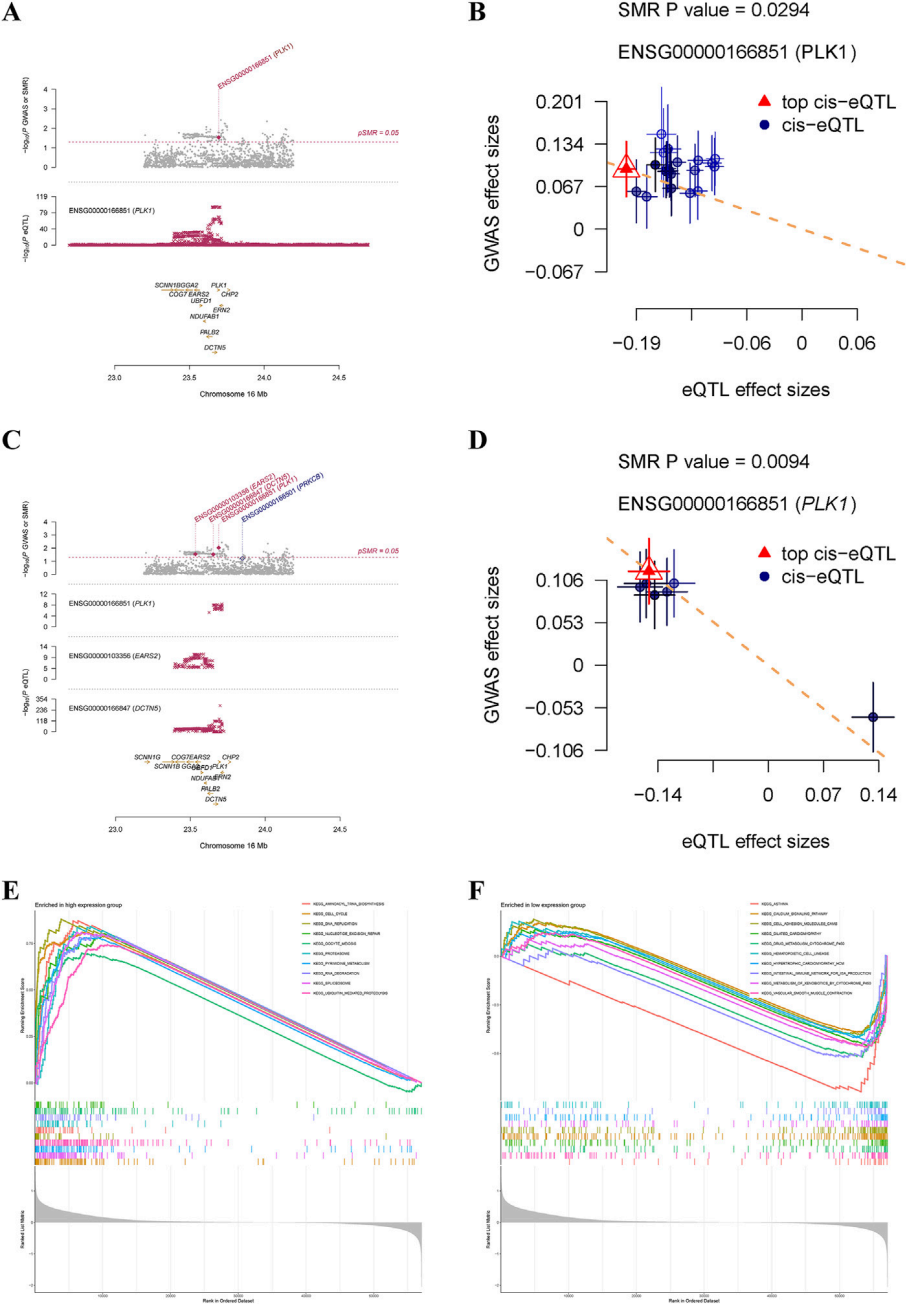
Summary-data-based Mendelian randomization analysis and single-gene gene set enrichment analysis. Locus zoom plot **(A)** and scatter plot **(B)** displayed causal effects between PLK1 and gastric cancer based on cis eQTLs from the eQTLGen consortium. Locus zoom plot **(C)** and scatter plot **(D)** displayed causal effects between PLK1 and gastric cancer based on cis eQTLs from the GTEx Consortium V8. Top 10 pathways significantly enriched in PLK1 high **(E)** and low **(F)** expression groups.

SMR analysis based on cis eQTLs from the eQTLGen Consortium indicated that genetically predicted higher levels of PLK1 increased the risk of GC (beta = 0.48; p value = 0.0294) (Figure 6A, B). The causal effect from PLK1 on GC was robust, as consistent evidence was obtained from SMR analysis performed using data from the GTEx Consortium (beta = 0.79; p value = 0.0094) (Figure 6C, D). Given that PLK1 was not only a causal gene for GC but also a key target of Diosgenin anti-GC, we further performed single-gene GSEA to elucidate its biological characteristics in GC. The results showed that cell cycle, DNA replication, oocyte meiosis, pyrimidine metabolism, the pathways promoting carcinogenesis were aberrantly activated in GC patients with high expression of PLK1 (Figure 6E). Conversely, in the PLK1 low-expression group, pathways including drug metabolism cytochrome P450, calcium signaling pathway, and cell adhesion molecules (CAMs) (Figure 6F). These findings further emphasize the role of PLK1 in the pathogenesis of GC.

### 3.6 Cell-type specificity expression in the GC tissue

**FIGURE 7:**
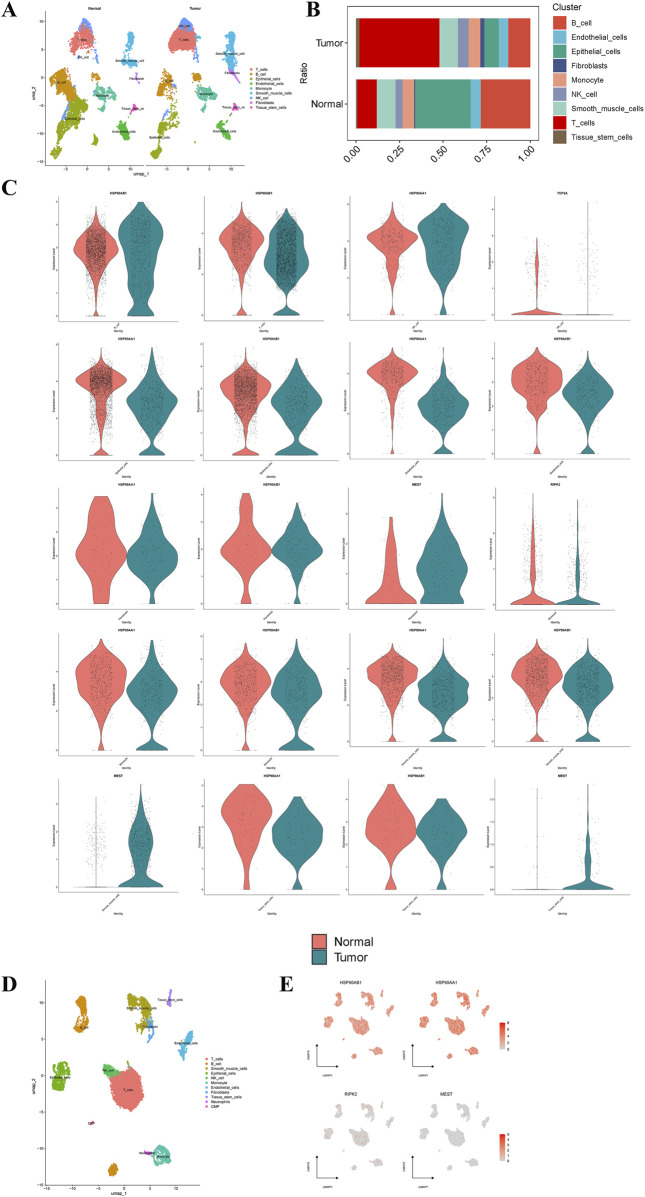
Single‐cell type expression in gastric tumor tissue for the anti-gastric cancer targets. **(A)** A total of 9 cell types were identified in normal and gastric cancer tissues. **(B)** Proportion of 9 cell types in normal and gastric cancer tissues. **(C)** Differential expression of the anti-gastric cancer targets of Diosgenin at the cell level in normal and gastric cancer tissues. **(D)** A total of 11 cell types were identified in gastric cancer tissues. **(E)** Specific expression of the anti-gastric cancer target of Diosgenin on cell types in gastric cancer tissues.

After quality control and processing of single-cell data, an expression matrix of 20,840 genes in 22,314 cells was obtained. This study first conducted single-cell sequencing analysis on GC and normal tissues. Following UMAP dimensionality reduction, 9 cell types were identified (Figure 7A). Compared to normal tissues, GC tissues exhibited a significant increase in T cells and a notable decrease in B cells and epithelial cells (Figure 7B). Single-cell analysis revealed that Diosgenin anti-GC targets HSP90AA1 and HSP90AB1 were significantly overexpressed in B cells and NK cells in GC tissues, respectively. Conversely, HSP90AA1 and HSP90AB1 expression was significantly reduced in endothelial cells, epithelial cells, fibroblasts, monocytes, smooth muscle cells, and tissue stem cells. MEST expression was significantly elevated in fibroblasts, smooth muscle cells, and tissue stem cells, while RIPK2 was significantly downregulated in monocytes, and TOP2A was significantly downregulated in NK cells (Figure 7C). When focusing solely on GC tissue for single-cell sequencing analysis, 11 cell types were identified (Figure 7D). HSP90AA1 and HSP90AB1 were significantly overexpressed in T cells and B cells in GC tissues, while MEST and RIPK2 were significantly overexpressed in smooth muscle cells and monocytes, respectively (Figure 7E). Overall, the dysregulation of T cells and B cells may play a critical role in GC, with HSP90AA1, HSP90AB1, and TOP2A emerging as key targets for Diosgenin-mediated immunomodulation.

### 3.7 Molecular docking

**FIGURE 8:**
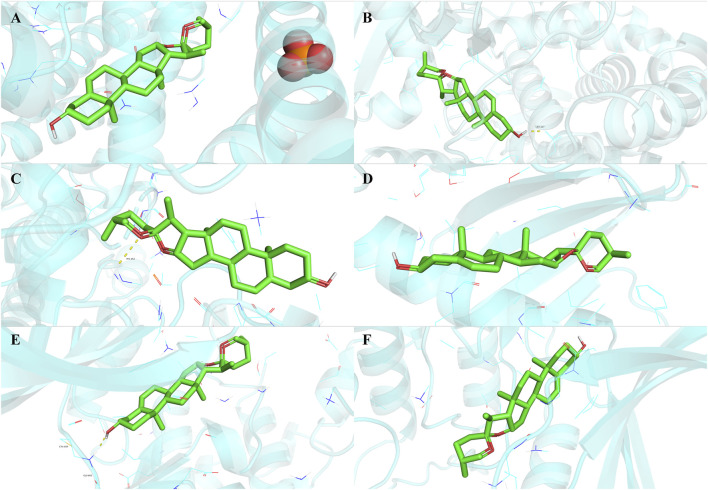
Molecular docking results of Diosgenin with anti-gastric cancer targets. **(A)** BLM. **(B)** CYP27B1. **(C)** CCNE1. **(D)** PLK4. **(E)** TTK. **(F)** CHEK1.

To validate the findings of network analysis, we obtained the small molecule ligand structure of Diosgenin (PubChem ID: 99474) from the PubChem Compound Database and performed molecular docking with the proteins encoded by the 19 anti-GC targets. The results demonstrated (Table 1) that Diosgenin exhibited strong binding affinity with all 19 protein receptors (binding energy < −5.0 kcal/mol). Further visualization of the top six ligand-protein receptor pairs with the strongest binding energies was performed using PyMOL (Figure 8).

**TABLE 1:** Molecular docking results of Diosgenin with anti-gastric cancer targets.

Protein receptors	PDB ID	Binding energy (kcal/mol)
BLM	5LUP	−10.4
CYP27B1	O15528	−10.3
CCNE1	7XQK	−9.8
PLK4	2N19	−9.7
TTK	2X9E	−9.7
CHEK1	2AYP	−9.5
CCNB1	2B9R	−9.4
TOP2A	1zxm	−9.3
BIRC5	1E31	−8.9
CDK1	6GU6	−8.8
PLK1	1Q4K	−8.6
HSP90AB1	1QZ2	−8.5
KIF11	3WPN	−8.2
RIPK2	5NG3	−8.2
CDC25C	2OJX	−8.0
HSP90AA1	1BYQ	−8.0
CCNA2	7LUO	−7.3
MEST	1SSL	−6.7
AURKB	4AF3	−6.6

### 3.8 Diosgenin inhibits proliferation, migration, and invasion of AGS cell via the MDM2/p53 signaling pathway

**FIGURE 9:**
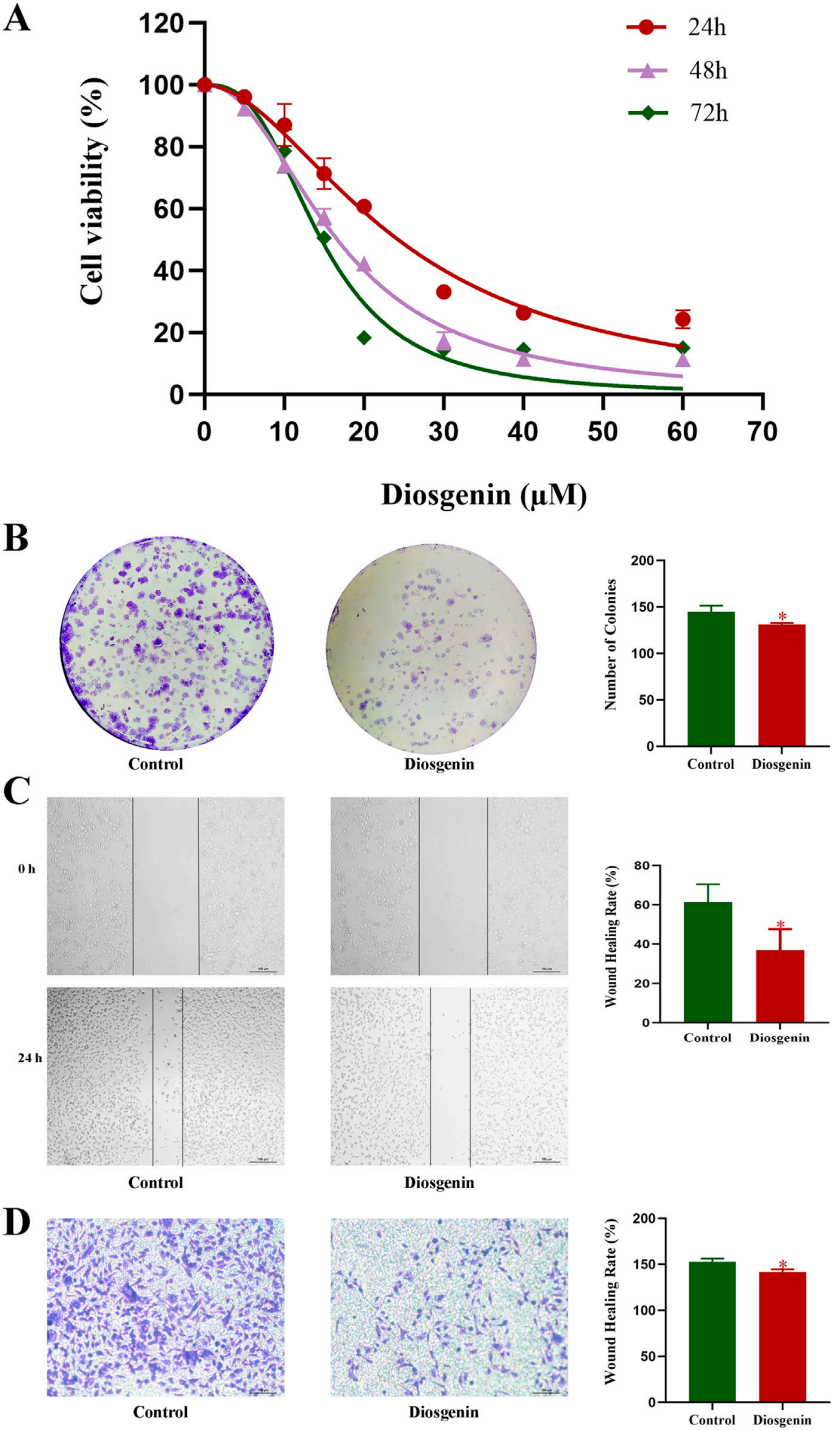
Diosgenin modulates the proliferation, migration, and invasive capabilities of GC cells. **(A)** Cell viability was measured using the CCK-8 assay. **(B)** Colony formation assays were performed to assess clonogenic potential. **(C)** Wound healing assays were used to evaluate cell migration. **(D)** Transwell assays were conducted to determine cell invasiveness. P value < 0.05*

To determine the appropriate experimental drug concentration, a CCK-8 assay was conducted. The results demonstrated that Diosgenin significantly inhibited the proliferation of AGS cells in a dose- and time-dependent manner (Figure 9A). The IC50 value of Diosgenin for AGS cells at 24 h was 28.336 ± 1.397 µM. Based on these findings, the IC20 value at 24 h was selected as the working concentration for subsequent experiments. Following 24-h treatment with Diosgenin, the colony formation assay revealed a significant reduction in the number of gastric cancer cell colonies (Figure 9B). Wound healing and Transwell invasion assays demonstrated that Diosgenin treatment for 24 h markedly suppressed the migratory and invasive capabilities of GC cells (Figure 9C, D).

**FIGURE 10:**
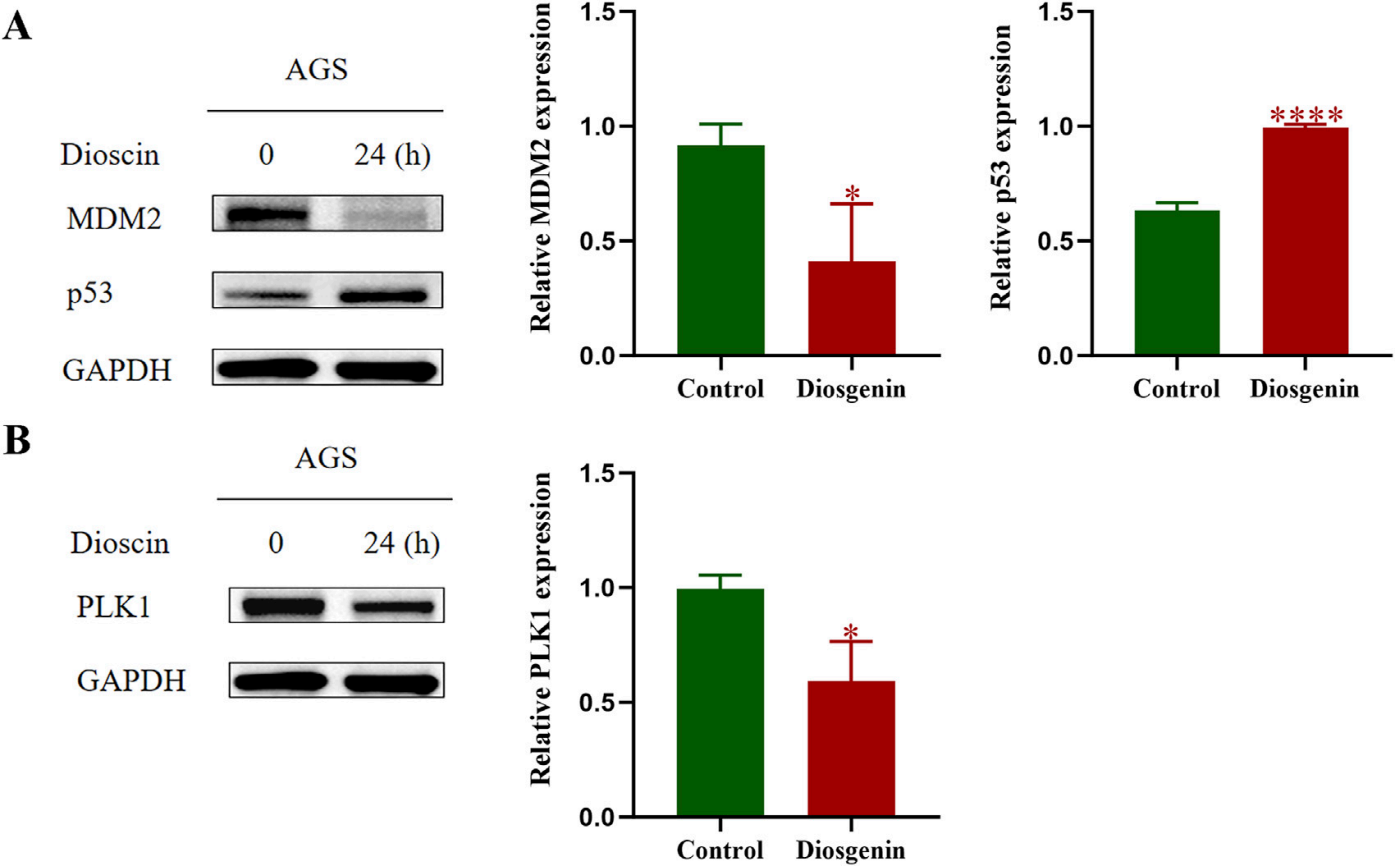
Western blot was performed to detect the protein expression levels of MDM2, p53, and PLK1 in GC cells. **(A)** Western blot results of MDM2, p53. **(B)** Western blot results of PLK1. P value < 0.05*, P value < 0.001***.

KEGG enrichment analysis of this work indicated that the p53 pathway may have the greatest potential in the anti-GC effects of Diosgenin. To further validate this hypothesis, Western blot analysis was performed, which revealed that after 24 h of Diosgenin treatment, MDM2 protein expression in AGS cells was significantly reduced, while p53 protein levels were markedly elevated compared to the untreated control group (Figure 10A). Additionally, previous SMR and GSEA analyses have highlighted PLK1 as a key risk gene for GC. Western blot results demonstrated that Diosgenin treatment for 24 h led to a significant reduction in PLK1 protein expression in AGS cells (Figure 10B).

## 4 Discussion

In this study, five (CDK1, CCNA2, TOP2A, CHEK1, and PLK1) core anti-GC targets of Diosgenin were identified, with four (CDK1, CCNA2, CHEK1, and PLK1) genes primarily involved in cell cycle regulation and one gene (TOP2A) associated with chemotherapy resistance. It is well known that cell cycle dysregulation is a hallmark of tumorigenesis. CDK1 is the only cyclin-dependent kinase essential for cell cycle progression in mammals, facilitating the G2/M and G1/S transitions as well as G1 progression (Santamaria et al., 2007; Enserink and Kolodner, 2010). Unrestricted cell proliferation, a hallmark of malignancy, is often driven by alterations in CDK1 activity. Over the past decades, numerous selective inhibitors or pan-inhibitors of CDK1 have been developed (Wang et al., 2023). Inhibiting CDK1 expression and activation effectively suppresses cancer cell activity in various cancers. Notably, some small molecules targeting CDK1 have been investigated in clinical trials, including for GC (Wang et al., 2023). A previous experimental study also confirmed that Diosgenin can induce G0/G1 phase arrest and apoptosis in AGS and SGC-7901 cells (Liu et al., 2020). The protein encoded by CCNA2 can activate cyclin-dependent kinase 2, playing a crucial role in facilitating the G1/S and G2/M transitions (Zhang et al., 2019). Recent studies have identified that CCNA2 may enhance cancer invasion, recurrence, metastasis, and chemoresistance (Fischer et al., 2016). Furthermore, research has reported that CCNA2 may contribute to the development and progression of various tumors by influencing EMT and metastasis (Bendris et al., 2012). Lee et al. (Bendris et al., 2012) demonstrated that CCNA2 expression is elevated in KRAS-mutant GC cell lines and primary tumors, increasing the sensitivity of GC patients to PLK1 inhibitors. GC cells with high CCNA2 expression are more dependent on PLK1 activity and exhibit increased mitotic index values. This dependency results in G2/M phase arrest and mitotic catastrophe, followed by apoptosis in response to PLK1 inhibitors. PLK1 is a serine/threonine protein kinase that is widely present in eukaryotic cells and is involved in the initiation, maintenance, and completion of mitosis. (Glover et al., 1998). PLK1 has been identified as a potential new target for cancer therapy, with several PLK1 kinase inhibitors developed as anti-cancer drugs currently under evaluation in clinical trials (Gutteridge et al., 2016). Research has shown that PLK1 promotes the metastasis and EMT of GC cells by regulating the AKT pathway (Cai et al., 2016). Inhibiting the HER2-SHCBP1-PLK1 axis can slow tumor cell mitosis and increase the sensitivity of GC to trastuzumab (Shi et al., 2021). Additionally, Otsu’s continuous follow-up of 207 GC patients revealed that those with high PLK1 expression had poorer prognoses (Otsu et al., 2016). Our study also confirmed that Diosgenin significantly inhibited PLK1 protein expression levels. Notably, Yousef et al. (2024) demonstrated that Diosgenin enhances the anti-hepatocarcinoma effects of PLK1 inhibitors and doxorubicin by downregulating the expression of PLK1 and PCNA. The protein encoded by CHEK1 belongs to the serine/threonine protein kinase family and mediates cell cycle arrest. When eukaryotic cells are exposed to factors causing DNA damage, such as ultraviolet light and reactive oxygen species, P53 mediates cell cycle arrest at the G1 phase, while Chk1 mediates cell cycle arrest at the S and G2 phases (Origanti et al., 2013). Therefore, mutations in TP53 result in the loss of functional p53, making cell cycle arrest entirely dependent on Chk1. For GC patients with TP53 mutations, CHEK1 is a highly promising therapeutic target. CHEK1 inhibitors prevent the activation of Chk1 by inhibiting its phosphorylation, thereby further suppressing the proliferation of TP53-mutant cancer cells (Geng et al., 2024). TOP2A is a key participant in the decatenation checkpoint. Defects in this checkpoint can lead to additional chromosomal imbalances in cancer cells, increasing tumor aggressiveness (Chen et al., 2015). Due to its crucial role, TOP2A has been identified as a primary target for developing anti-tumor chemotherapeutic drugs that inhibit its DNA-cleaving activity, such as doxorubicin, etoposide, and mitoxantrone (Liu et al., 2019). TOP2A is overexpressed in various malignancies and is associated with chemotherapy resistance, abnormal cell proliferation, aneuploidy, tumor recurrence, and reduced overall survival (Liu et al., 2019). High expression of TOP2A is currently believed to be associated with chemotherapy resistance in various malignancies, including breast cancer (Liu et al., 2023) and colorectal cancer (Zhu et al., 2021). The correlation between TOP2A and chemotherapy resistance in GC has not yet been reported. Interestingly, studies have found that EF1-mediated upregulation of TOP2A promotes the survival, migration, and invasion of GC cells while inhibiting their apoptosis (Chen et al., 2022). Our team previously discovered that Diosgenin can significantly inhibit the expression of EF1 (Li et al., 2019). This suggests that Diosgenin may also downregulate TOP2A expression, thereby inhibiting GC progression. Overall, these biomarkers could be important targets for future research on the anti-GC mechanisms of Diosgenin.

p53 is a tumor suppressor protein, often referred to as the “guardian of the genome,” that plays a critical regulatory role in stress responses such as DNA damage induced by ionizing radiation. It mediates a series of proteins involved in cell cycle regulation, checkpoint control, DNA repair, and apoptosis. Given that p53-dependent cell cycle arrest and apoptosis are detrimental to the growth and survival of normal cells, the activity of p53 is tightly regulated in healthy cells and tissues. Approximately 30% of GC have been observed to exhibit p53 mutations or deletions (Soussi et al., 2006). MDM2 is a major negative regulator of p53, inhibiting its activity by binding to and disrupting the interaction between p53 and its target gene promoters (Oliner et al., 1992). Amplification of MDM2 has been reported in 10% of GC (Sun et al., 2004). Moreover, in numerous tumors, downregulation of MDM2 has been shown to suppress tumor growth or induce apoptosis (Shangary and Wang, 2009; Huang et al., 2013). Thus, restoring p53 signaling through MDM2-p53 inhibitors has emerged as a highly promising therapeutic strategy for treating p53 wild-type cancer. *In vitro* experiments by Wang et al. revealed that Triptolide induced apoptosis in GC cells by inhibiting MDM2 overexpression, but the pathway was not dependent on p53 (Wang et al., 2014). The MDM2/p53 pathway was also found to mediate the progression of *Helicobacter* pylori-induced GC. Deng et al. (Deng et al., 2023) discovered that N4-acetylcytidine (ac4C) mRNA modification and its acetyltransferase, N-acetyltransferase 10 (NAT10), were significantly overexpressed in human GC tissues. Further *in vitro* and *in vivo* experiments demonstrated that *H. pylori* infection contributed to the upregulation of NAT10, leading to MDM2 overexpression and subsequent p53 degradation, thereby promoting GC progression. Another study found that APG-115, a novel MDM2/p53 small molecule inhibitor, enhanced *in vitro* and *in vivo* anti-GC effects when combined with radiation therapy (Yi et al., 2018). This was achieved by inhibiting MDM2 protein expression, thereby relieving p53 suppression. In this study, we confirmed that Diosgenin exhibited strong inhibitory effects on AGS cell proliferation, colony formation, migration, and invasion. The MDM2/p53 pathway may represent a potential mechanism underlying the anti-GC effects of Diosgenin.

This study provides a comprehensive and multi-omics analysis to elucidate the anti-GC mechanisms of Diosgenin, integrating bioinformatics, experimental validation, and molecular docking. Key strengths include: Multi-omics Approach: The integration of gene expression data, weighted gene co-expression network analysis (WGCNA), and single-cell sequencing offers a systems-level understanding of Diosgenin’s therapeutic potential. Identification of Core Targets: Five critical anti-GC targets (CDK1, CCNA2, TOP2A, CHEK1, and PLK1) were identified, with a focus on their roles in cell cycle regulation and chemotherapy resistance, providing mechanistic insights into Diosgenin’s anti-tumor effects. Experimental Validation: *In vitro* experiments confirmed that Diosgenin inhibits GC cell proliferation, migration, and invasion, while Western blot analysis validated its modulation of key proteins (MDM2, p53, and PLK1). Pathway Analysis: KEGG enrichment revealed that Diosgenin likely exerts its anti-GC effects through critical pathways such as the p53 signaling pathway, cell cycle, and immune regulation, highlighting its multi-faceted therapeutic potential. Clinical Relevance: The study identified targets with high diagnostic value (AUC: 0.909–0.984), suggesting their potential as biomarkers for GC diagnosis. Despite these strengths, the study has several limitations: Lack of *In Vivo* Validation: The findings are primarily based on *in vitro* experiments and bioinformatics predictions. *In vivo* studies are needed to confirm the efficacy and safety of Diosgenin in animal models or clinical settings. Mechanistic Depth: While the study identifies key targets and pathways, the precise molecular mechanisms by which Diosgenin modulates these targets remain to be fully elucidated. Predictive Nature of In Silico Tools: The target predictions derived from the database are still speculative and lack experimental verification. These tools are prone to false positives, particularly for small molecule, which often exhibit non-specific binding. Their reliance on structural similarity further compounds this uncertainty, as shared scaffolds do not guarantee shared bioactivity. Notably, blind docking risks overestimating promiscuous interactions, as seen with metabolites like Diosgenin. Inherent Bias in Computational Outcomes: Such studies invariably generate results, but these often conflate noise with signal. Without orthogonal assays (e.g., binding affinity measurements or functional studies), the biological relevance of predicted targets remains unsubstantiated. Cherry-picking statistically favorable but biologically unverified outcomes exacerbates this issue. Pan-Assay Interference Concerns: Computational hits may include pan-assay interfering compounds (PAINS), which exhibit nonspecific activity *in vitro* but lack therapeutic utility. Rigorous experimental follow-up is essential to exclude such artifacts. Clinical Applicability: The translation of these findings into clinical applications requires further investigation, including pharmacokinetic studies and clinical trials to assess the therapeutic potential of Diosgenin in GC patients.

Overall, Diosgenin is a promising natural metabolite that may be important for retarding the progression of GC. It is necessary to further confirm the anti-GC value of Diosgenin *in vivo* or clinical studies in the future.

## Data Availability

The original contributions presented in the study are included in the article/supplementary material, further inquiries can be directed to the corresponding authors.

## References

[B1] Aran D. Looney A. P. Liu L. Wu E. Fong V. Hsu A. (2019). Reference-based analysis of lung single-cell sequencing reveals a transitional profibrotic macrophage. Nat. Immunol. 20 (2), 163–172. 10.1038/s41590-018-0276-y 30643263 PMC6340744

[B2] Bendris N. Arsic N. Lemmers B. Blanchard J. M. Cyclin A. (2012). Cyclin A2, Rho GTPases and EMT. Small GTPases 3 (4), 225–228. 10.4161/sgtp.20791 22735340 PMC3520886

[B3] Bray F. Laversanne M. Sung H. Ferlay J. Siegel R. L. Soerjomataram I. (2024). Global cancer statistics 2022: GLOBOCAN estimates of incidence and mortality worldwide for 36 cancers in 185 countries. CA Cancer J. Clin. 74 (3), 229–263. 10.3322/caac.21834 38572751

[B4] Butler A. Hoffman P. Smibert P. Papalexi E. Satija R. (2018). Integrating single-cell transcriptomic data across different conditions, technologies, and species. Nat. Biotechnol. 36 (5), 411–420. 10.1038/nbt.4096 29608179 PMC6700744

[B5] Cai X. P. Chen L. D. Song H. B. Zhang C. X. Yuan Z. W. Xiang Z. X. (2016). PLK1 promotes epithelial-mesenchymal transition and metastasis of gastric carcinoma cells. Am. J. Transl. Res. 8 (10), 4172–4183. 27830001 PMC5095310

[B6] Chen T. Sun Y. Ji P. Kopetz S. Zhang W. (2015). Topoisomerase IIα in chromosome instability and personalized cancer therapy. Oncogene 34 (31), 4019–4031. 10.1038/onc.2014.332 25328138 PMC4404185

[B7] Chen Y. U. Yu Y. Lv M. Shi Q. Li X. (2022). E2F1-mediated up-regulation of TOP2A promotes viability, migration, and invasion, and inhibits apoptosis of gastric cancer cells. J. Biosci. 47, 84. 10.1007/s12038-022-00322-2 36550695

[B8] Davies N. M. Holmes M. V. Davey Smith G. (2018). Reading Mendelian randomisation studies: a guide, glossary, and checklist for clinicians. BMJ 362, k601. 10.1136/bmj.k601 30002074 PMC6041728

[B9] Deng M. Zhang L. Zheng W. Chen J. Du N. Li M. (2023). Helicobacter pylori-induced NAT10 stabilizes MDM2 mRNA via RNA acetylation to facilitate gastric cancer progression. J. Exp. Clin. Cancer Res. 42 (1), 9. 10.1186/s13046-022-02586-w 36609449 PMC9817303

[B10] Enserink J. M. Kolodner R. D. (2010). An overview of Cdk1-controlled targets and processes. Cell Div. 5, 11. 10.1186/1747-1028-5-11 20465793 PMC2876151

[B11] Fischer M. Quaas M. Steiner L. Engeland K. (2016). The p53-p21-DREAM-CDE/CHR pathway regulates G2/M cell cycle genes. Nucleic Acids Res. 44 (1), 164–174. 10.1093/nar/gkv927 26384566 PMC4705690

[B12] Geng H. Qian R. Zhong Y. Tang X. Zhang X. Zhang L. (2024). Leveraging synthetic lethality to uncover potential therapeutic target in gastric cancer. Cancer Gene Ther. 31 (2), 334–348. 10.1038/s41417-023-00706-y 38040871

[B13] Glover D. M. Hagan I. M. Tavares A. A. (1998). Polo-like kinases: a team that plays throughout mitosis. Genes Dev. 12 (24), 3777–3787. 10.1101/gad.12.24.3777 9869630

[B14] Grau J. Grosse I. Keilwagen J. (2015). PRROC: computing and visualizing precision-recall and receiver operating characteristic curves in R. Bioinformatics 31 (15), 2595–2597. 10.1093/bioinformatics/btv153 25810428 PMC4514923

[B15] Gu L. Zheng H. Zhao R. Zhang X. Wang Q. (2021). Diosgenin inhibits the proliferation of gastric cancer cells via inducing mesoderm posterior 1 down-regulation-mediated alternative reading frame expression. Hum. Exp. Toxicol. 40 (12_Suppl. l), S632–S645. 10.1177/09603271211053292 34806916

[B16] Gutteridge R. E. Ndiaye M. A. Liu X. Ahmad N. (2016). Plk1 inhibitors in cancer therapy: from laboratory to clinics. Mol. Cancer Ther. 15 (7), 1427–1435. 10.1158/1535-7163.MCT-15-0897 27330107 PMC4936921

[B17] Huang M. Zhang H. Liu T. Tian D. Gu L. Zhou M. (2013). Triptolide inhibits MDM2 and induces apoptosis in acute lymphoblastic leukemia cells through a p53-independent pathway. Mol. Cancer Ther. 12 (2), 184–194. 10.1158/1535-7163.MCT-12-0425 23243057 PMC3570632

[B18] Jesus M. Martins A. P. Gallardo E. Silvestre S. (2016). Diosgenin: recent highlights on pharmacology and analytical methodology. J. Anal. Methods Chem. 2016, 4156293. 10.1155/2016/4156293 28116217 PMC5225340

[B19] Khanal P. Patil V. S. Bhandare V. V. Patil P. P. Patil B. M. Dwivedi P. S. R. (2022). Systems and *in vitro* pharmacology profiling of Diosgenin against breast cancer. Front. Pharmacol. 13, 1052849. 10.3389/fphar.2022.1052849 36686654 PMC9846155

[B20] Kumar V. Ramnarayanan K. Sundar R. Padmanabhan N. Srivastava S. Koiwa M. (2022). Single-cell Atlas of lineage States, tumor microenvironment, and subtype-specific expression programs in gastric cancer. Cancer Discov. 12 (3), 670–691. 10.1158/2159-8290.CD-21-0683 34642171 PMC9394383

[B21] Kurki M. I. Karjalainen J. Palta P. Sipila T. P. Kristiansson K. Donner K. M. (2023). FinnGen provides genetic insights from a well-phenotyped isolated population. Nature 613 (7944), 508–518. 10.1038/s41586-022-05473-8 36653562 PMC9849126

[B22] Langfelder P. Horvath S. (2008). WGCNA: an R package for weighted correlation network analysis. BMC Bioinforma. 9, 559. 10.1186/1471-2105-9-559 PMC2631488 19114008

[B23] Li F. Fernandez P. P. Rajendran P. Hui K. M. Sethi G. (2010). Diosgenin, a steroidal saponin, inhibits STAT3 signaling pathway leading to suppression of proliferation and chemosensitization of human hepatocellular carcinoma cells. Cancer Lett. 292 (2), 197–207. 10.1016/j.canlet.2009.12.003 20053498

[B24] Li S. Y. Shang J. Mao X. M. Fan R. Li H. Q. Li R. H. (2021). Diosgenin exerts anti-tumor effects through inactivation of cAMP/PKA/CREB signaling pathway in colorectal cancer. Eur. J. Pharmacol. 908, 174370. 10.1016/j.ejphar.2021.174370 34324855

[B25] Li X. Yu D. Wang Q. Chen Y. Jiang H. (2024). Elucidating the molecular mechanisms of pterostilbene against cervical cancer through an integrated bioinformatics and network pharmacology approach. Chem. Biol. Interact. 396, 111058. 10.1016/j.cbi.2024.111058 38761877

[B26] Li Y. Zhu L. Shi F. Li R. Chen X. Zheng Z. (2019). Modified Liangfu granule exhibits anti-cancer effects in gastric cancer by regulating apoptosis-related proteins and genes. J. Traditional Chin. Med. Sci. 6 (04), 325–330. 10.1016/j.jtcms.2019.11.002

[B27] Liu S. Rong G. Li X. Geng L. Zeng Z. Jiang D. (2020). Diosgenin and GSK126 produce synergistic effects on epithelial-mesenchymal transition in gastric cancer cells by mediating EZH2 via the Rho/ROCK signaling pathway. Onco Targets Ther. 13, 5057–5067. 10.2147/OTT.S237474 32606728 PMC7292386

[B28] Liu T. Zhang H. Yi S. Gu L. Zhou M. (2019). Mutual regulation of MDM4 and TOP2A in cancer cell proliferation. Mol. Oncol. 13 (5), 1047–1058. 10.1002/1878-0261.12457 30672125 PMC6487731

[B29] Liu Y. Yu K. Zhang K. Niu M. Chen Q. Liu Y. (2023). O-GlcNAcylation promotes topoisomerase IIα catalytic activity in breast cancer chemoresistance. EMBO Rep. 24 (7), e56458. 10.15252/embr.202256458 37249035 PMC10328065

[B30] Mao Z. J. Tang Q. J. Zhang C. A. Qin Z. F. Pang B. Wei P. K. (2012). Anti-proliferation and anti-invasion effects of diosgenin on gastric cancer BGC-823 cells with HIF-1α shRNAs. Int. J. Mol. Sci. 13 (5), 6521–6533. 10.3390/ijms13056521 22754381 PMC3382793

[B31] Morris G. M. Huey R. Olson A. J. (2008). Using AutoDock for ligand-receptor docking. Curr. Protoc. Bioinforma. 8. 10.1002/0471250953.bi0814s24 19085980

[B32] Newman A. M. Liu C. L. Green M. R. Gentles A. J. Feng W. Xu Y. (2015). Robust enumeration of cell subsets from tissue expression profiles. Nat. Methods 12 (5), 453–457. 10.1038/nmeth.3337 25822800 PMC4739640

[B33] Newman D. J. Cragg G. M. (2016). Natural products as sources of new drugs from 1981 to 2014. J. Nat. Prod. 79 (3), 629–661. 10.1021/acs.jnatprod.5b01055 26852623

[B34] Nogales C. Mamdouh Z. M. List M. Kiel C. Casas A. I. Schmidt H. (2022). Network pharmacology: curing causal mechanisms instead of treating symptoms. Trends Pharmacol. Sci. 43 (2), 136–150. 10.1016/j.tips.2021.11.004 34895945

[B35] Oliner J. D. Kinzler K. W. Meltzer P. S. George D. L. Vogelstein B. (1992). Amplification of a gene encoding a p53-associated protein in human sarcomas. Nature 358 (6381), 80–83. 10.1038/358080a0 1614537

[B36] Origanti S. Cai S. R. Munir A. Z. White L. S. Piwnica-Worms H. (2013). Synthetic lethality of Chk1 inhibition combined with p53 and/or p21 loss during a DNA damage response in normal and tumor cells. Oncogene 32 (5), 577–588. 10.1038/onc.2012.84 22430210 PMC3381958

[B37] Otsu H. Iimori M. Ando K. Saeki H. Aishima S. Oda Y. (2016). Gastric cancer patients with high PLK1 expression and DNA aneuploidy correlate with poor prognosis. Oncology 91 (1), 31–40. 10.1159/000445952 27245623

[B38] Oun R. Moussa Y. E. Wheate N. J. (2018). The side effects of platinum-based chemotherapy drugs: a review for chemists. Dalton Trans. 47 (19), 6645–6653. 10.1039/c8dt00838h 29632935

[B39] Santamaria D. Barriere C. Cerqueira A. Hunt S. Tardy C. Newton K. (2007). Cdk1 is sufficient to drive the mammalian cell cycle. Nature 448 (7155), 811–815. 10.1038/nature06046 17700700

[B40] Sethi G. Shanmugam M. K. Warrier S. Merarchi M. Arfuso F. Kumar A. P. (2018). Pro-apoptotic and anti-cancer properties of diosgenin: a comprehensive and critical review. Nutrients 10 (5), 645. 10.3390/nu10050645 29783752 PMC5986524

[B41] Shangary S. Wang S. (2009). Small-molecule inhibitors of the MDM2-p53 protein-protein interaction to reactivate p53 function: a novel approach for cancer therapy. Annu. Rev. Pharmacol. Toxicol. 49, 223–241. 10.1146/annurev.pharmtox.48.113006.094723 18834305 PMC2676449

[B42] Shi W. Zhang G. Ma Z. Li L. Liu M. Qin L. (2021). Hyperactivation of HER2-SHCBP1-PLK1 axis promotes tumor cell mitosis and impairs trastuzumab sensitivity to gastric cancer. Nat. Commun. 12 (1), 2812. 10.1038/s41467-021-23053-8 33990570 PMC8121856

[B43] Soussi T. Asselain B. Hamroun D. Kato S. Ishioka C. Claustres M. (2006). Meta-analysis of the p53 mutation database for mutant p53 biological activity reveals a methodologic bias in mutation detection. Clin. Cancer Res. 12 (1), 62–69. 10.1158/1078-0432.CCR-05-0413 16397025

[B44] Sun L. P. Jiang N. J. Fu W. Xue Y. X. Zhao Y. S. (2004). Relationship between gastric cancer and gene amplification of p14 and mdm2. Ai Zheng 23 (1), 36–39. 14720371

[B45] Vosa U. Claringbould A. Westra H. J. Bonder M. J. Deelen P. Zeng B. (2021). Large-scale cis- and trans-eQTL analyses identify thousands of genetic loci and polygenic scores that regulate blood gene expression. Nat. Genet. 53 (9), 1300–1310. 10.1038/s41588-021-00913-z 34475573 PMC8432599

[B46] Wang B. Y. Cao J. Chen J. W. Liu Q. Y. (2014). Triptolide induces apoptosis of gastric cancer cells via inhibiting the overexpression of MDM2. Med. Oncol. 31 (11), 270. 10.1007/s12032-014-0270-7 25280518

[B47] Wang Q. Bode A. M. Zhang T. (2023). Targeting CDK1 in cancer: mechanisms and implications. NPJ Precis. Oncol. 7 (1), 58. 10.1038/s41698-023-00407-7 37311884 PMC10264400

[B48] Wei L. Sun J. Zhang N. Zheng Y. Wang X. Lv L. (2020). Noncoding RNAs in gastric cancer: implications for drug resistance. Mol. Cancer 19 (1), 62. 10.1186/s12943-020-01185-7 32192494 PMC7081551

[B49] Wu T. Hu E. Xu S. Chen M. Guo P. Dai Z. (2021). clusterProfiler 4.0: a universal enrichment tool for interpreting omics data. Innov. (Camb) 2 (3), 100141. 10.1016/j.xinn.2021.100141 PMC8454663 34557778

[B50] Yi H. Yan X. Luo Q. Yuan L. Li B. Pan W. (2018). A novel small molecule inhibitor of MDM2-p53 (APG-115) enhances radiosensitivity of gastric adenocarcinoma. J. Exp. Clin. Cancer Res. 37 (1), 97. 10.1186/s13046-018-0765-8 29716622 PMC5930807

[B51] Yousef E. H. El-Mesery M. E. Habeeb M. R. Eissa L. A. (2024). Diosgenin potentiates the anticancer effect of doxorubicin and volasertib via regulating polo-like kinase 1 and triggering apoptosis in hepatocellular carcinoma cells. Naunyn Schmiedeb. Arch. Pharmacol. 397 (7), 4883–4894. 10.1007/s00210-023-02894-8 38165424

[B52] Yu G. Wang L. G. Yan G. R. He Q. Y. (2015). DOSE: an R/Bioconductor package for disease ontology semantic and enrichment analysis. Bioinformatics 31 (4), 608–609. 10.1093/bioinformatics/btu684 25677125

[B53] Zhang S. Tischer T. Barford D. (2019). Cyclin A2 degradation during the spindle assembly checkpoint requires multiple binding modes to the APC/C. Nat. Commun. 10 (1), 3863. 10.1038/s41467-019-11833-2 31455778 PMC6712056

[B54] Zhu C. Zhang L. Zhao S. Dai W. Xu Y. Zhang Y. (2021). UPF1 promotes chemoresistance to oxaliplatin through regulation of TOP2A activity and maintenance of stemness in colorectal cancer. Cell Death Dis. 12 (6), 519. 10.1038/s41419-021-03798-2 34021129 PMC8140095

